# Comprehensive evaluation of frost mulberry leaves of 21 mulberry varieties

**DOI:** 10.3389/fpls.2025.1592493

**Published:** 2025-10-08

**Authors:** Jie Tian, Bingxiang Liu, Zhitong Fan, Jingyan Yang, Yibo Wu, Xinyuan Tian, Chenyang Jia, Haichao Wen, Bingying Zhang, Hongjiao Li

**Affiliations:** ^1^ College of Forestry, Hebei Agricultural University, Baoding, China; ^2^ Yunwuping Forest Farm in Jiangjin District, Chongqing, China; ^3^ Food Science and Technology College, Hebei Agricultural University, Baoding, China; ^4^ Hebei Academy of Fine Arts, Shijiazhuang, China; ^5^ Hebei Hongya Mountain State-Owned Forest Farm, Baoding, China

**Keywords:** mulberry, frost mulberry leaf, nutritional quality, antioxidant capacity, principal component analysis, entropy weight method, grey relational analysis

## Abstract

**Introduction:**

Mulberry, a medicinal and edible plant, exhibits significant pharmacological effects, including immune enhancement, blood lipid and glucose reduction, and tumor inhibition, due to the abundance of nutrients and bioactive components in its leaves. However, traditional mulberry breeding has primarily focused on developing high-yielding, high-quality, stress-resistant, and mechanizable leaf-type mulberry varieties, leading to a relatively uniform product output. With the increasing diversification of mulberry applications, the criteria for variety selection and quality evaluation have become more stringent.

**Methods:**

In this study, 21 mulberry varieties were selected as experimental materials to evaluate the variations in nutritional quality, functional components, and antioxidant properties of frost mulberry leaves across different varieties. Principal component analysis (PCA) was employed to determine key evaluation indicators. Subsequently, the entropy‐weight method was employed to assign weights to these core indicators, and grey relational analysis (GRA) was applied, to comprehensively assess the overall quality of frost mulberry leaves from the 21 varieties.

**Results:**

The results indicate that, in terms of nutritional quality, 'Da 10' exhibited the highest free amino acid content; however, its low soluble sugar content negatively affected leaf texture. In contrast, 'Jiang Mi Guo Sang', 'Lv Shen Zi 2', and 'Ju Shen' demonstrated superior overall performance. Regarding functional components, 'Tang 10', 'Da Yi Bai', 'Da Bai E', and 'Lv Shen Zi 1' excelled across all indicators. Concerning antioxidant capacity, 'Da Bai E', 'Jiang Mi Guo Sang', 'He Lan Sang', and 'Ju Shen' ranked highly, while 'Da 10', 'Da Yi Bai', 'Hong Guo 1', and 'Da Bai E' exhibited higher enzyme activity. PCA identified 10 core indicators related to nutritional quality, functional components, and antioxidant capacity, with a cumulative variance contribution rate of 83.752%. The entropy weight method was then used to assign weights to these indicators, and the final grey relational degree, calculated using grey system theory, ranged from 0.476 to 0.785.

**Conclusion:**

This study demonstrates that the varieties 'Da Bai E', 'Da Yi Bai', 'Da 10', 'He Lan Sang', and 'Tang 10' exhibit superior overall quality and high nutritional value, providing theoretical support for the subsequent selection, cultivation, and utilization of mulberry varieties.

## 1 Introduction

Mulberry (*Morus alba* L.), a perennial woody plant of the family Moraceae and genus Morus, is renowned for its high adaptability and broad distribution (Zhong et al., 2022). It is an economically significant forest tree species in China, primarily found in the Yangtze River Basin, the Pearl River Delta, the Yellow-Huai region, and the Yunnan-Guizhou-Sichuan area (Hu et al., 2024). Mulberry possesses significant medicinal and economic value, as its roots, branches, leaves, and fruits contain abundant nutrients, serving as essential raw materials for traditional Chinese medicine and various food products. The National Health Commission of China has classified mulberry as a plant resource with both medicinal and edible properties (Hao et al., 2022).

Mulberry leaves, also known as home mulberry, Jingxing mulberry, mulberry fruit tree, and yellow mulberry leaves, constitute the primary yield of mulberry trees and are widely cultivated (Tejaswini et al., 2025). Recognized by the National Health Commission of China as both a medicinal and edible food source, mulberry leaves contain an array of nutrients and bioactive compounds, including proteins, vitamins, polysaccharides, flavonoids, mineral elements, and alkaloids. They exhibit multiple health benefits, such as lowering blood sugar and lipid levels and possessing antiviral properties (Ma et al., 2022). Mulberry leaves contain 8-12% crude fiber, 3-5% crude fat, and 8-12% crude ash (Yan et al., 2023) and are rich in vitamins, triterpenoids, volatile oils, choline, organic acids, minerals, pigments, and other bioactive components. The average protein mass fraction is 22.42%, with crude protein content reaching up to 14.43% (Wang et al., 2012). Key bioactive compounds consist of flavonoids, polyphenols, alkaloids, polysaccharides, phytosterols, and γ-aminobutyric acid. Liu et al. (2025) analyzed 30 mulberry leaf samples from 17 varieties across three mulberry species, testing 37 quality indicators, including starch and soluble sugar. Their results revealed significant differences among varieties, with starch content ranging from 64.99 to 86.50 g/kg and water-soluble sugar content from 61.20 to 137.99 g/kg. Similarly, Yu et al. (2018) examined the nutritional and phytochemical components of branches and leaves from 19 mulberry varieties in China, reporting total phenol and flavonoid contents of 8.76–20.26 mg/g and 21.36–56.41 mg/g dry weight, respectively.

In addition to their traditional role in sericulture, mulberry leaves have been extensively utilized in traditional and modern medicine. Since ancient times, they have been traditionally used for liver and kidney nourishment, blood circulation enhancement, thirst quenching, upper burner clearance, and lung heat alleviation. The Shen Nong Ben Cao Jing first documented their properties, stating that they could “remove cold and heat and induce sweating,” while the Compendium of Materia Medica noted their potential to “improve eyesight and promote hair growth.” Modern medical research has demonstrated that mulberry leaves can lower blood sugar levels due to their bioactive components, including alkaloids, flavonoids, and polysaccharides (Lin et al., 2022). Flavonoids exhibit anti-inflammatory and lipid-lowering effects, while alkaloids, including 1-DNJ and its derivatives, have been shown to lower blood sugar levels and exert antiviral and anti-inflammatory properties (Cai et al., 2016; Liao et al., 2024). Additionally, mulberry leaf polysaccharides contribute to glucose and lipid metabolism regulation, mitigating insulin resistance (Dai et al., 2024). The medicinal properties of mulberry leaves may reduce the risk of chronic diseases, offering potential therapeutic applications. A wide range of mulberry leaf-based products have been developed, including health products, food, beverages, and condiments. Commercially available products include mulberry leaf tea (Yang et al., 2024), mulberry-flavored sauces, vinegar, tofu, biscuits, noodles, and liquors. James et al. (2024) investigated the therapeutic potential of mulberry leaf tea in health management, while Lu et al (Gan et al., 2024) employed ultrafine grinding techniques to substitute flour with mulberry leaf powder in biscuit production.

Frost mulberry leaves (also known as frost-hit or winter mulberry leaves) are harvested from mulberry trees (*Moraceae*) after exposure to frost. They possess significant medicinal value, are rich in bioactive compounds such as flavonoids, and exhibit pharmacological effects such as dispelling wind-heat. These leaves are abundant, readily accessible, and possess high application potential. Frost-treated mulberry leaves are widely utilized in traditional and modern medicine (Sugiyama et al., 2017). They are documented in numerous classical Chinese medical texts, where most recorded mulberry leaves were collected after frost. The Chinese Pharmacopoeia’s earlier editions also list frost-treated mulberry leaves. This phenomenon suggests that frost-treated mulberry leaves possess superior medicinal value (Xu et al., 2021). Research indicates that the levels of bioactive compounds in mulberry leaves fluctuate seasonally, with flavonoid content significantly increasing after frost (Yu et al., 2017). Frost-treated mulberry leaves are notable for their combined medicinal and nutritional benefits, along with their abundance and accessibility (Wu et al., 2025).

Despite numerous studies on mulberry quality analysis, most research has primarily concentrated on fruit-bearing mulberry varieties. However, evaluation criteria for mulberry leaves remain largely independent, and the challenge of integrating various assessment methods into a comprehensive evaluation framework requires further investigation. Establishing appropriate evaluation criteria is essential for developing a standardized assessment system. Over time, plant variety evaluation methods have progressed from qualitative assessments to quantitative analyses, shifting from single-indicator evaluations to comprehensive multi-criteria assessment frameworks. The current evaluation framework does not incorporate a comprehensive system that simultaneously considers five or more key indicators, necessitating further in-depth investigation. By analyzing key indicators of mulberry leaf varieties, superior cultivars can be identified, facilitating the efficient utilization of mulberry trees and enhancing the economic benefits of the mulberry industry. This study presents an innovative and comprehensive evaluation of 21 mulberry varieties to overcome the limitations of single-method assessments. Principal component analysis (PCA) and correlation analysis (CA) were employed to identify the key quality indicators of mulberry leaves. Additionally, the entropy weight method-grey relational analysis (EWM-GRA) approach was applied to identify the most suitable mulberry varieties for Hebei Province based on their comprehensive quality scores. This study aims to support the development of ecologically sustainable, three-dimensional mulberry cultivation in Hebei Province by providing a scientific basis tailored to local conditions. Additionally, it offers a methodological reference for multi-indicator assessments of other economic forest species.

## 2 Materials and methods

### 2.1 Experimental materials

The study utilized 21 mulberry germplasm resources (**Table 1**), all sourced from the mulberry germplasm repository at Hebei Agricultural University. Three trees from each variety were randomly selected for observation. The trees were three years old, spaced 1.5 m × 3.0 m apart. All trees exhibited uniform growth under standardized management conditions and were free from diseases and pests. Leaves were collected on 24 October 2021, immediately after the Frost’s Descent solar term. Leaves positioned between the 5th and 7th nodes from the middle of the branches were harvested. Only healthy and vigorously growing plants were chosen for sampling. Collected leaf samples were transported to the laboratory, washed with distilled water to remove surface debris, and subsequently rinsed with deionized water. The leaves were blotted dry using absorbent paper, placed in an oven at 105°C for 15 minutes to inactivate enzymatic activity, and subsequently dried at 65°C until a constant weight was achieved. The dried samples were pulverized, passed through a 50-mesh sieve, and stored at -80°C for subsequent analysis.

**Table 1:** List of test materials.

No.	Material name	Introduced
X1	Ri Ben Guo Sang	Zhenjiang City, Jiangsu Province
X2	Jiang Mi Guo Sang	Dongguang County, Hebei Province
X3	Tian Sang 202	Zhenjiang City, Jiangsu Province
X4	Hong Guo 1	Xianyang City, Shaanxi Province
X5	Bai Shen 2	Zhenjiang City, Jiangsu Province
X6	Lv Shen Zi	Xiajin County, Shandong Province
X7	Lv Shen Zi 1	Xiajin County, Shandong Province
X8	Lv Shen Zi 2	Xiajin County, Shandong Province
X9	Hei Zhen Zhu	Maoming City, Guangdong Province
X10	Ji Gui Hua	Cangzhou City, Hebei Province
X11	Gui Hua Mi	Qian’an City, Hebei Province
X12	Xiao Bai E	Dongguang County, Hebei Province
X13	Da Bai E	Dongguang County, Hebei Province
X14	Da Yi Bai	Li County, Hebei Province
X15	Su Bai Shen	Zhenjiang City, Jiangsu Province
X16	Feng Guo Sang	Zhenjiang City, Jiangsu Province
X17	He Lan Sang	Zhenjiang City, Jiangsu Province
X18	Ju Shen	Zhenjiang City, Jiangsu Province
X19	Da 10	Zhenjiang City, Jiangsu Province
X20	Tang 10	Guangzhou City, Guangdong Province
X21	Da Ma Ya	Linqing City, Shandong Province

### 2.2 Experimental methods

#### 2.2.1 Photosynthetic pigments of mulberry leaves

Photosynthetic pigment contents, including chlorophyll a, chlorophyll b, carotenoids, and total chlorophyll, were measured using the ethanol extraction method (Li et al., 2020).

#### 2.2.2 Determination of functional components of mulberry leaves

The content of free amino acids was determined using the ninhydrin colorimetric method (Anantharaman et al., 2017). The titratable acid content was measured using the phenolphthalein indicator colorimetric method (Wang et al., 2024). The soluble sugar content was analyzed using the anthrone colorimetric method (Reis et al., 2015). The soluble protein content was quantified using the Coomassie Brilliant Blue G-250 colorimetric method (Bradford, 1976).

The calibration curve for determining free amino acids is y=9.154x-0.9307 (R^2^ = 0.9935), the calibration curve for determining soluble sugars is y=0.0063x-0.0052 (R^2^ = 0.9989), and the calibration curve for determining soluble proteins is y=0.0049x-0.001 (R^2^ = 0.9979).

#### 2.2.3 Evaluation of antioxidant quality of mulberry leaves

The tannin content was determined using the Folin-Ciocalteu colorimetric method (Peraman et al., 2016). The flavonoid content was assessed via the aluminum chloride (AlCl_3_) colorimetric method (Liu et al., 2017), while the polyphenol content was quantified using the Folin-Ciocalteu colorimetric method (Nezam et al., 2021). Anthocyanin content was analyzed using the pH differential method (Handayani et al., 2024), whereas resveratrol content was determined through ethanol (C_2_H_5_OH) extraction (Zhu et al., 2022). Chlorogenic acid content was measured using the ferrous ion (Fe^2+^) colorimetric method (Irena et al., 2019). The determination of ascorbic acid content is carried out by titration (Wu et al., 2023).

Among them, the calibration curve for determining tannin is y=15.205x+0.0512 (R^2^ = 0.9934), the calibration curve for determining flavonoids is y=6.5536x+0.002 (R^2^ = 0.9981), and the calibration curve for determining polyphenols is y=17.95x+0.1427 (R^2^ = 0.9936). The calibration curve for the determination of resveratrol was y=100.56x-0.0024 (R2 = 0.9996), and the calibration curve for the determination of chlorogenic acid was y=1.906x-0.0.1583 (R2 = 0.9929).

#### 2.2.4 Antioxidant quality of the mulberry leaves

The ability to scavenge DPPH and ABTS radicals, as well as the iron-reducing power, was assessed following the methods described by Fatiha et al. (2022). A precisely weighed 0.1 g sample was extracted using 50% ethanol (C_2_H_5_OH) at a solid-to-liquid ratio of 1:50. Ultrasonic extraction was performed for 1 hour, after which the extract was diluted to a final volume of 10.0 mL for subsequent analysis. For DPPH radical scavenging activity, 0.5 mL of the extract was mixed with 2.0 mL of DPPH reagent, thoroughly shaken, and incubated in the dark for 30 minutes. The absorbance was then measured at 517 nm. Similarly, for ABTS radical scavenging activity, 0.5 mL of the extract was combined with 2.0 mL of ABTS reagent, mixed thoroughly, and incubated in the dark for 6 minutes before measuring the absorbance at 734 nm. Iron-reducing power was determined by mixing 0.5 mL of the extract with 1.0 mL of distilled water and 1.8 mL of TPTZ working solution, shaking the mixture well, and allowing it to react in the dark for 10 minutes. The absorbance was recorded at 593 nm. Superoxide dismutase (SOD) activity was measured using the NBT photochemical reduction method (Giannopolitis and Ries, 1977). Peroxidase (POD) activity was determined using the guaiacol method (Samara et al., 2019). Catalase (CAT) activity was assessed via the hydrogen peroxide decomposition method (Havir and Mchale, 1987). Polyphenol oxidase (PPO) activity was analyzed using the catechol method (Ross and Matthew, 2014), while lipoxygenase (LOX) activity was measured according to Ikram et al. (2024). Malondialdehyde (MDA) content was determined using the thiobarbituric acid (TBA) colorimetric method.

Among them, the calibration curve for determining DPPH is y=-0.0052x+0.9358 (R^2^ = 0.996), and the calibration curve for determining ABTS is y=-0.0065x+0.5572 (R^2^ = 0.9995), the calibration curve for determining the ability of iron to reduce oxygen is y=0.0073x+0.0551 (R^2^ = 0.9994).

### 2.3 Data processing and analysis

Data processing and summarization were conducted using Microsoft Excel 2016, while data analysis was performed in SPSS 26.0, employing Duncan’s new multiple range test to determine significant differences. The comprehensive evaluation method incorporated principal component analysis (PCA) to identify core evaluation indicators. Based on these indicators, the entropy weight method (EWM) was applied to assign weights, and grey relational analysis (GRA) was utilized to conduct a comprehensive evaluation of different mulberry varieties.

The experimental data were normalized and used as comparison sequences, denoted as X*_i_* (I = 0, 1, …, 21), representing patterns I to XXI. The optimal values for each indicator were selected as the optimal sequence X_0_ (*k*), with a discrimination coefficient *ρ* = 0.5, where *k* = {1, 2, …, *m*}. The correlation coefficients *S* between each sequence and the reference sequence were calculated using the following Formula (1):


(1) $$S_{i}\left(k\right)=\frac{min_{i}min_{k}\left|x_{0}\left(k\right)-x_{i}\left(k\right)\right|+\rho \cdot max_{i}max_{k}\left|x_{0}\left(k\right)-x_{i}\left(k\right)\right|}{\left|x_{0}\left(k\right)-x_{i}\left(k\right)\right|+\rho \cdot max_{i}max_{k}\left|x_{0}\left(k\right)-x_{i}\left(k\right)\right|}$$

The entropy weight method (EWM) was applied to determine the weight (*W*) of each evaluation indicator, and the weighted correlation coefficient (*R*) was calculated using the following Formula (2):


(2) $$R_{0i}=\frac{1}{m}\sum_{k=1}^{m}W_{k}\cdot S\left(k\right)$$

## 3 Results and analysis

### 3.1 Differences in photosynthetic pigment content of frost mulberry leaves among different varieties

The measurements of chlorophyll a, chlorophyll b, carotenoids, and total chlorophyll content in the frost leaves of different mulberry varieties are presented in **Table 2**. Within the same column, different lowercase letters following the data indicate significant differences among varieties based on Duncan’s test (*P* < 0.05), whereas identical letters denote no significant differences (*P* > 0.05).

**Table 2:** Photosynthetic pigment content of different varieties of frost mulberry leaves (mg/g).

Varieties	Chlorophyll a content	Chlorophyll b content	Carotenoid content	Chlorophyll content
Ri Ben Guo Sang	1.20 ± 0.09bcd	0.53 ± 0.04a	0.25 ± 0.03cdefg	1.72 ± 0.13bc
Jiang Mi Guo Sang	1.26 ± 0.03bc	0.46 ± 0.01ab	0.23 ± 0.02fghi	1.72 ± 0.03bc
Tian Sang 202	0.78 ± 0.07ij	0.26 ± 0.03ef	0.18 ± 0.02ij	1.04 ± 0.08i
Hong Guo 1	1.15 ± 0.13cde	0.47 ± 0.05ab	0.30 ± 0.05abcd	1.62 ± 0.18cde
Bai Shen 2	1.13 ± 0.09cde	0.36 ± 0.03cd	0.28 ± 0.03bcdef	1.49 ± 0.11def
Lv Shen Zi	1.48 ± 0.06a	0.50 ± 0.01a	0.34 ± 0.02a	1.99 ± 0.06a
Lv Shen Zi 1	0.87 ± 0.14ghij	0.27 ± 0.05ef	0.19 ± 0.03hij	1.13 ± 0.19hi
Lv Shen Zi 2	1.02 ± 0.14defg	0.38 ± 0.04bc	0.26 ± 0.03bcdefg	1.40 ± 0.18ef
Hei Zhen Zhu	1.21 ± 0.06bcd	0.40 ± 0.02bc	0.31 ± 0.02ab	1.61 ± 0.08cde
Ji Gui Hua	1.10 ± 0.01cdef	0.35 ± 0.01cde	0.26 ± 0.02bcdefg	1.45 ± 0.01ef
Gui Hua Mi	1.37 ± 0.03ab	0.50 ± 0.05a	0.30 ± 0.01abc	1.87 ± 0.06ab
Xiao Bai E	1.38 ± 0.26ab	0.45 ± 0.10ab	0.29 ± 0.05abcde	1.82 ± 0.36ab
Da Bai E	1.03 ± 0.06defg	0.33 ± 0.01cde	0.22 ± 0.02ghi	1.35 ± 0.06fg
Da Yi Bai	1.08 ± 0.03cdefg	0.39 ± 0.02bc	0.24 ± 0.02efgh	1.47 ± 0.05def
Su Bai Shen	0.95 ± 0.08efghi	0.31 ± 0.02cde	0.24 ± 0.03defg	1.27 ± 0.10fgh
Feng Guo Sang	0.54 ± 0.07k	0.21 ± 0.01f	0.09 ± 0.01k	0.75 ± 0.08j
He Lan Sang	0.80 ± 0.16hij	0.26 ± 0.07ef	0.15 ± 0.03j	1.05 ± 0.23ghi
Ju Shen	1.13 ± 0.24cde	0.46 ± 0.11ab	0.29 ± 0.05abcde	1.58 ± 0.34bcd
Da 10	1.01 ± 0.15defgh	0.34 ± 0.06cde	0.28 ± 0.03bcdef	1.35 ± 0.20ef
Tang 10	0.89 ± 0.08fghij	0.40 ± 0.05bc	0.19 ± 0.03ij	1.30 ± 0.12fgh
Da Ma Ya	0.72 ± 0.10jk	0.28 ± 0.05def	0.17 ± 0.03j	1.00 ± 0.15i

Values are expressed as mean ± standard deviation. Different lowercase letters indicate significant differences in Duncan test (*P* < 0.05).

Chlorophyll a content in frost mulberry leaves from 21 mulberry varieties ranged from 0.54 to 1.48 mg/g, with an average of 1.05 mg/g. The variety ‘Lv Shen Zi’ exhibited the highest chlorophyll a content (1.48 mg/g), which did not significantly differ from ‘Xiao Bai E’, ‘Gui Hua Mi’, and ‘Ju Shen’, but was significantly higher than that of other varieties. No significant differences in chlorophyll a content were observed among ‘Ri Ben Guo Sang’, ‘Ju Shen’, ‘Hong Guo 1’, ‘Da 10’, and ‘Bai Shen 2’. ‘Feng Guo Sang’ exhibited the lowest chlorophyll a content (0.54 mg/g), which was significantly lower than that of all other varieties.

Chlorophyll b content in frost mulberry leaves ranged from 0.21 to 0.53 mg/g, with an average of 0.38 mg/g. ‘Ri Ben Guo Sang’ exhibited the highest chlorophyll b content (0.53 mg/g), which did not significantly differ from ‘Lv Shen Zi’, ‘Gui Hua Mi’, ‘Ju Shen’, ‘Xiao Bai E’, and ‘Hong Guo 1’, but was significantly higher than that of other varieties. The chlorophyll b content in ‘Feng Guo Sang’ was not significantly different from that in ‘Lv Shen Zi 1’ and ‘Tian Sang 202’, but was significantly lower than that in other varieties, measuring only 0.21 mg/g.

Carotenoid content ranged from 0.09 to 0.34 mg/g, with an average of 0.24 mg/g. ‘Lv Shen Zi’ exhibited the highest carotenoid content (0.34 mg/g), which did not significantly differ from ‘Hei Zhen Zhu’, ‘Xiao Bai E’, and five other varieties, but was significantly higher than that of the remaining varieties. ‘Feng Guo Sang’ exhibited the lowest carotenoid content (0.09 mg/g).

Total chlorophyll content ranged from 0.75 to 1.99 mg/g, with an average of 1.43 mg/g. ‘Lv Shen Zi’ exhibited the highest total chlorophyll content (1.99 mg/g), which did not significantly differ from ‘Xiao Bai E’ and ‘Gui Hua Mi’, but was significantly higher than that of other varieties. ‘Feng Guo Sang’ exhibited the lowest total chlorophyll content (0.75 mg/g). A high chlorophyll content may enhance photosynthetic efficiency (Andrea et al., 2023), thereby indirectly increasing biomass accumulation in frost mulberry leaves. Furthermore, a high carotenoid content (0.34 mg/g) may enhance antioxidant capacity (Nurlaela et al., 2018).

### 3.2 Differences in nutritional quality of frost mulberry leaves among different varieties

The results of moisture content, free amino acids, soluble sugar, soluble protein, and tannin content measurements in frost leaves from different mulberry varieties are presented in **Table 3**.

**Table 3:** Differences in nutritional quality of frosted mulberry leaves in different varieties of mulberry trees.

Varieties	Water content (%)	Free amino acid content(mg/g)	Soluble sugar content (%)	Soluble protein content(µg/g)
Ri Ben Guo Sang	58.71 ± 2.34abcd	108.11 ± 15d	17.93 ± 2.63hijk	19.76 ± 0.26cde
Jiang Mi Guo Sang	51.58 ± 2.62fgh	138.41 ± 6.47b	27.49 ± 1.98ab	24.88 ± 1.14a
Tian Sang 202	58.27 ± 2.63abcd	101.9 ± 7.07de	22.62 ± 0.15cde	22.40 ± 0.83b
Hong Guo 1	58.02 ± 2.23abcd	102.71 ± 5.05de	11.09 ± 1.38l	20.45 ± 0.32bcde
Bai Shen 2	60.12 ± 1.51ab	104.63 ± 6.41de	25.44 ± 0.70bc	19.84 ± 1.50cde
Lv Shen Zi	61.24 ± 2.88a	121.07 ± 5.97c	26.44 ± 1.82ab	20.49 ± 0.66bcd
Lv Shen Zi 1	58.86 ± 0.77abc	110.31 ± 3.72d	17.27 ± 1.34ijk	21.34 ± 1.59bc
Lv Shen Zi 2	50.65 ± 2.23ghi	124.08 ± 3.78c	20.31 ± 0.67defgh	20.86 ± 2.07bcd
Hei Zhen Zhu	56.82 ± 1.72bcde	80.60 ± 2.32gh	19.32 ± 0.03fghij	20.02 ± 0.53bcde
Ji Gui Hua	56.70 ± 3.03bcde	84.16 ± 8.63g	23.11 ± 1.01cd	16.72 ± 0.36f
Gui Hua Mi	56.51 ± 2.29bcde	95.35 ± 9.77ef	18.23 ± 1.22ghijk	20.69 ± 1.09bcd
Xiao Bai E	57.78 ± 0.71abcd	102.32 ± 5.68de	22.63 ± 1.90cde	18.39 ± 1.36def
Da Bai E	53.37 ± 1.31efg	128.51 ± 8.05c	21.14 ± 1.51defg	17.89 ± 1.81ef
Da Yi Bai	47.41 ± 1.00i	87.59 ± 3.84fg	21.93 ± 2.23def	19.63 ± 1.66cde
Su Bai Shen	53.41 ± 2.41efg	96.94 ± 4.71ef	20.62 ± 2.31defgh	16.57 ± 2.54f
Feng Guo Sang	49.02 ± 0.24hi	73.15 ± 2.75h	16.32 ± 1.71jk	18.87 ± 0.63cdef
He Lan Sang	55.58 ± 1.39cde	147.39 ± 5.16b	28.48 ± 2.33a	19.92 ± 2.02bcde
Ju Shen	54.92 ± 0.61def	165.74 ± 4.78a	19.79 ± 1.85efghi	20.51 ± 1.18bcd
Da 10	56.17 ± 1.42cde	171.42 ± 8.34a	16.64 ± 0.97jk	18.89 ± 0.85cdef
Tang 10	50.68 ± 2.38ghi	90.83 ± 3.21fg	15.73 ± 1.12k	17.89 ± 0.57ef
Da Ma Ya	48.22 ± 2.26hi	89.82 ± 4.37fg	17.88 ± 1.52hijk	20.21 ± 1.29bcde

Different lowercase letters indicate significant differences in Duncan test (P<0.05).

The moisture content of frost mulberry leaves among the 21 varieties ranged from 47.41% to 61.24%, with an average of 54.95%. The variety ‘Lv Shen Zi’ exhibited the highest moisture content (61.24%), which was not significantly different from ‘Bai Shen 2’, ‘Lv Shen Zi 1’, ‘Ri Ben Guo Sang’, ‘Tian Sang 202’, ‘Hong Guo 1’, and ‘Xiao Bai E’, but was significantly higher than that of the remaining varieties. Conversely, ‘Da Yi Bai’ displayed the lowest moisture content (47.41%), which was not significantly different from ‘Tang 10’, ‘Lv Shen Zi 2’, ‘Feng Guo Sang’, and ‘Da Ma Ya’, but was significantly lower than that of the other varieties.

The free amino acid content ranged from 73.15 mg/g to 171.42 mg/g, with an average of 110.72 mg/g. ‘Da 10’ exhibited the highest free amino acid content (171.42 mg/g), which was not significantly different from ‘Ju Shen’ but was significantly higher than that of the other varieties. In contrast, ‘Feng Guo Sang’ had the lowest free amino acid content (73.15 mg/g), which was not significantly different from ‘Hei Zhen Zhu’ but was significantly lower than that of the remaining varieties.

The soluble sugar content varied between 11.09% and 28.48%, with an average of 20.50%. ‘He Lan Sang’ exhibited the highest soluble sugar content (28.48%), which may be attributed to the accumulation of sweet-tasting compounds in medicinal mulberry leaves. A high soluble sugar content enhances the palatability of frost mulberry leaves and serves as an energy source for the synthesis of secondary metabolites (Liu et al., 2024). No significant differences in soluble sugar content were observed among ‘Ji Gui Hua’, ‘Xiao Bai E’, ‘Tian Sang 202’, ‘Da Yi Bai’, ‘Da Bai E’, ‘Su Bai Shen’, and ‘Lv Shen Zi 2’. The lowest soluble sugar content (11.09%) was recorded in ‘Hong Guo 1’.

The soluble protein content ranged from 16.57 µg/g to 24.88 µg/g, with an average of 19.82 µg/g. ‘Jiang Mi Guo Sang’ had the highest soluble protein content (24.88 µg/g), which was significantly higher than that of all other varieties. Soluble proteins contain essential amino acids and bioactive peptides that contribute to various health benefits, including antibacterial, antioxidant, and anti-inflammatory properties (De Taddeo et al., 2023).

### 3.3 Differences in functional components of frost mulberry leaves among different varieties

The results of the flavonoid, polyphenol, anthocyanin, resveratrol, chlorogenic acid, and ascorbic acid content measurements in frost leaves from different mulberry varieties are presented in **Table 4**.

**Table 4:** Differences in the functional composition of fruits in different varieties of mulberry trees.

Varieties	Flavonoid content (mg/g)	Polyphenol content (mg/g)	Resveratrol content (mg/g)	Resveratrol content (mg/g)	Ascorbic acid content (mg/100g)	Tannin content (mg/g)
Ri Ben Guo Sang	87.15 ± 6.92cdef	47.43 ± 4.62jk	1.66 ± 0.17cde	19.62 ± 0.48j	179.58 ± 11.96b	1.44 ± 0.11efg
Jiang Mi Guo Sang	72.50 ± 4.89fghij	92.89 ± 11.76cd	1.79 ± 0.20bcd	44.01 ± 1.91cd	68.66 ± 5.19ij	1.32 ± 0.14fg
Tian Sang 202	100.30 ± 3.42bc	42.05 ± 3.74k	1.13 ± 0.11ghi	21.04 ± 1.61ij	130.23 ± 3.23cde	0.85 ± 0.13i
Hong Guo 1	62.10 ± 4.11jk	37.57 ± 5.12kl	0.95 ± 0.19i	29.11 ± 4.44gh	140.01 ± 5.69cd	1.46 ± 0.34ef
Bai Shen 2	70.55 ± 8.86ghij	72.67 ± 4.42fg	1.84 ± 0.21bc	39.20 ± 5.87de	133.82 ± 11.85cd	0.90 ± 0.12hi
Lv Shen Zi	53.15 ± 3.40k	73.80 ± 1.71fg	1.77 ± 0.14bcd	32.39 ± 3.11fg	151.06 ± 10.07c	1.85 ± 0.21cd
Lv Shen Zi 1	78.76 ± 6.23defghi	108.70 ± 13.32ab	1.96 ± 0.18abc	48.35 ± 1.26abc	81.66 ± 14.95hi	2.15 ± 0.04b
Lv Shen Zi 2	106.71 ± 9.21ab	61.13 ± 10.78hi	1.18 ± 0.08ghi	21.16 ± 1.06ij	108.43 ± 7.26efg	1.63 ± 0.16de
Hei Zhen Zhu	68.46 ± 2.00hijk	27.27 ± 3.19l	1.41 ± 0.20defg	26.76 ± 6.67ghi	144.05 ± 26.09cd	1.34 ± 0.05efg
Ji Gui Hua	61.46 ± 14.77jk	85.37 ± 2.21de	2.27 ± 0.29a	48.17 ± 1.87abc	126.85 ± 12.43def	1.16 ± 0.03gh
Gui Hua Mi	88.96 ± 1.34cde	70.69 ± 0.86fgh	1.14 ± 0.17ghi	36.60 ± 1.37ef	150.61 ± 20.19c	2.31 ± 0.29ab
Xiao Bai E	64.65 ± 10.15ijk	82.29 ± 4.16def	2.06 ± 0.04ab	46.33 ± 0.89bc	88.87 ± 3.14ghi	2.10 ± 0.30bc
Da Bai E	80.92 ± 5.48defgh	111.44 ± 8.38a	2.27 ± 0.30a	50.87 ± 2.41ab	55.25 ± 15.22j	2.38 ± 0.09ab
Da Yi Bai	78.20 ± 15.26defghi	115.48 ± 3.02a	2.16 ± 0.26ab	53.39 ± 3.33a	102.48 ± 16.22gh	2.13 ± 0.08bc
Su Bai Shen	90.66 ± 3.09cd	54.81 ± 0.90ij	1.26 ± 0.05fghi	26.76 ± 2.80ghi	175.47 ± 13.01b	1.40 ± 0.05efg
Feng Guo Sang	63.22 ± 7.73ijk	41.18 ± 6.18k	1.59 ± 0.02cdef	24.64 ± 6.72hij	179.54 ± 2.86b	0.90 ± 0.12hi
He Lan Sang	73.78 ± 5.92efghij	100.14 ± 7.21bc	1.79 ± 0.25bcd	43.79 ± 1.66cd	202.44 ± 5.22a	1.16 ± 0.19gh
Ju Shen	86.06 ± 8.50cdefg	57.96 ± 2.23ij	0.96 ± 0.25hi	26.6 ± 4.86ghi	105.62 ± 9.80fg	1.37 ± 0.14efg
Da 10	120.04 ± 8.37a	78.61 ± 8.06ef	1.35 ± 0.20efgh	28.03 ± 6.40gh	125.59 ± 9.87def	1.63 ± 0.03de
Tang 10	84.84 ± 4.89cdefg	89.48 ± 6.89cde	2.31 ± 0.24a	42.29 ± 4.20cde	129.99 ± 20.97cde	2.46 ± 0.05a
Da Ma Ya	80.77 ± 17.40defgh	63.84 ± 5.38ghi	1.03 ± 0.22ghi	37.44 ± 1.47def	101.18 ± 4.83gh	1.15 ± 0.10gh

Different lowercase letters indicate significant differences in Duncan test (P<0.05).

The flavonoid content in frost mulberry leaves across 21 varieties ranged from 53.15 mg/g to 120.04 mg/g, with an average of 79.68 mg/g. The highest flavonoid content was observed in ‘Da 10’ (120.04 mg/g), which was not significantly different from ‘Lv Shen Zi 2’ but was significantly higher than that of other varieties. No significant differences were found in the flavonoid content among ‘Su Bai Shen’, ‘Gui Hua Mi’, ‘Ri Ben Guo Sang’, ‘Ju Shen’, ‘Tang 10’, ‘Da Bai E’, ‘Da Ma Ya’, and ‘Lv Shen Zi 1’. The lowest flavonoid content was recorded in ‘Lv Shen Zi’ (53.15 mg/g), which was not significantly different from ‘Hei Zhen Zhu’, ‘Xiao Bai E’, ‘Feng Guo Sang’, ‘Hong Guo 1’, and ‘Ji Gui Hua’, but was significantly lower than that of the remaining varieties.

The polyphenol content ranged from 27.27 mg/g to 115.48 mg/g, with an average of 72.13 mg/g. ‘Da Yi Bai’ exhibited the highest polyphenol content (115.48 mg/g), which was not significantly different from ‘Da Bai E’. Polyphenols possess anti-inflammatory and blood sugar-lowering properties, making varieties with high polyphenol content ideal raw materials for functional food development (Macaluso et al., 2024). The polyphenol content in ‘Xiao Bai E’ was not significantly different from that of ‘Da 10’, ‘Lv Shen Zi’, ‘Bai Shen 2’, and ‘Gui Hua Mi’. The lowest polyphenol content was recorded in ‘Hei Zhen Zhu’ (27.27 mg/g), which was not significantly different from ‘Hong Guo 1’.

The resveratrol content varied between 0.95 mg/g and 2.31 mg/g, with an average of 1.61 mg/g. ‘Tang 10’ had the highest resveratrol content (2.31 mg/g), which was not significantly different from ‘Ji Gui Hua’, ‘Da Bai E’, and ‘Tang 10’. In contrast, ‘Hong Guo 1’ exhibited the lowest resveratrol content (0.95 mg/g), which was not significantly different from ‘Su Bai Shen’, ‘Lv Shen Zi 2’, ‘Gui Hua Mi’, ‘Tian Sang 202’, ‘Da Ma Ya’, and ‘Ju Shen’.

The chlorogenic acid content ranged from 19.62 mg/g to 53.39 mg/g, with an average of 35.55 mg/g. ‘Da Yi Bai’ exhibited the highest chlorogenic acid content (53.39 mg/g), which was not significantly different from ‘Da Bai E’, ‘Lv Shen Zi 1’, and ‘Ji Gui Hua’ but was significantly higher than that of other varieties. The lowest chlorogenic acid content was found in ‘Ri Ben Guo Sang’ (19.62 mg/g), which was not significantly different from ‘Feng Guo Sang’, ‘Lv Shen Zi 2’, and ‘Tian Sang 202’.

Ascorbic acid plays a crucial role in maintaining immune system function, promoting wound healing, and mitigating oxidative stress. Its antioxidant properties help protect against free radical damage and are essential for skin health (Bisma et al., 2021). The ascorbic acid content in frost mulberry leaves ranged from 55.25 mg/100g to 202.44 mg/100g, with an average of 127.68 mg/100g. ‘He Lan Sang’ exhibited the highest ascorbic acid content (202.44 mg/100g), which was significantly higher than that of all other varieties.

The tannin content ranged from 0.85 mg/g to 2.46 mg/g, with an average of 1.58 mg/g. ‘Tang 10’ exhibited the highest tannin content (2.46 mg/g), which was not significantly different from ‘Da Bai E’ and ‘Gui Hua Mi’ but was significantly higher than that of other varieties. The lowest tannin content was recorded in ‘Tian Sang 202’ (0.85 mg/g), which was not significantly different from ‘Bai Shen 2’ and ‘Feng Guo Sang’ but was significantly lower than that of the remaining varieties.

### 3.4 Differences in antioxidant qualities of frost mulberry leaves among different varieties

#### 3.4.1 Differences in antioxidant capacity of frost mulberry leaves among different varieties

The results of the measurements of DPPH and ABTS radical scavenging capacities, iron-reducing power, and malondialdehyde (MDA) content in frost leaves from different mulberry varieties are presented in **Table 5**.

**Table 5:** Differences in the oxidation resistance of mulberry leaves in different varieties of mulberry trees.

Varieties	DPPH free radical scavenging ability µmol/L(Trolox)	ABTS free radical scavenging ability	Iron reduction capability (µmol/L) Trolox	MDA content (µmol/g)
Ri Ben Guo Sang	24.12 ± 3.69bcdef	19.06 ± 0.62abc	79.38 ± 12.31jk	25.92 ± 3.91ghi
Jiang Mi Guo Sang	29.82 ± 0.77a	17.23 ± 0.79defg	140.97 ± 11.03de	48.12 ± 5.02bc
Tian Sang 202	22.93 ± 2.52bcdef	16.44 ± 2.46efghij	94.88 ± 9.16hijk	37.45 ± 6.42cdefg
Hong Guo 1	26.07 ± 1.02abcd	16.59 ± 1.10efghi	76.30 ± 4.51jk	40.31 ± 3.04cde
Bai Shen 2	12.12 ± 3.71i	14.73 ± 0.23j	152.11 ± 8.10d	40.11 ± 7.90cde
Lv Shen Zi	16.67 ± 4.97h	15.30 ± 0.56hij	128.69 ± 0.82ef	27.52 ± 1.16fghi
Lv Shen Zi 1	27.98 ± 1.11ab	19.65 ± 0.06ab	122.23 ± 8.11efg	27.54 ± 5.82fghi
Lv Shen Zi 2	27.45 ± 3.59ab	20.10 ± 0.56a	87.45 ± 8.41ijk	20.46 ± 1.74i
Hei Zhen Zhu	19.07 ± 2.27fgh	19.18 ± 0.29abc	71.07 ± 4.26k	32.46 ± 4.06defgh
Ji Gui Hua	17.18 ± 2.89gh	14.84 ± 0.35ij	160.98 ± 20.41cd	34.30 ± 1.81defg
Gui Hua Mi	19.97 ± 3.27efgh	15.56 ± 1.33ghij	129.77 ± 15.06ef	36.66 ± 9.13defg
Xiao Bai E	27.11 ± 1.93abc	18.12 ± 0.68bcde	96.35 ± 9.81hij	27.01 ± 6.14ghi
Da Bai E	25.13 ± 3.07abcde	15.98 ± 0.29ghij	255.08 ± 17.46a	38.99 ± 2.79cdef
Da Yi Bai	19.31 ± 2.91fgh	15.98 ± 1.32ghij	191.11 ± 29.12b	43.20 ± 6.95cd
Su Bai Shen	19.39 ± 3.11fgh	19.19 ± 0.38abc	99.40 ± 7.82ghij	21.36 ± 2.63hi
Feng Guo Sang	21.51 ± 3.33defgh	16.78 ± 1.11efgh	113.42 ± 15.32fgh	31.21 ± 1.20efghi
He Lan Sang	18.95 ± 2.96fgh	18.66 ± 0.27abc	181.96 ± 10.68bc	56.93 ± 11.73b
Ju Shen	27.79 ± 0.79ab	19.83 ± 0.10ab	78.68 ± 6.30jk	36.40 ± 2.34defg
Da 10	22.21 ± 0.55cdefg	17.82 ± 0.98cdef	125.40 ± 19.70ef	74.89 ± 6.52a
Tang 10	23.46 ± 0.30bcdef	16.12 ± 1.18fghij	158.58 ± 6.66d	32.66 ± 4.01defgh
Da Ma Ya	23.53 ± 2.26bcdef	16.73 ± 1.46efgh	106.39 ± 9.64fghi	48.48 ± 12.84bc

Different lowercase letters indicate significant differences in Duncan test (P<0.05).

The ability to scavenge DPPH radicals is a key indicator of antioxidant potential, as it reflects the capacity to neutralize free radicals and mitigate oxidative stress-related chronic diseases, such as cardiovascular diseases and cancer (Liu and Zhang, 2024). The DPPH radical scavenging capacity of frost mulberry leaves across 21 varieties ranged from 12.12 to 29.82 µmol/L (Trolox equivalents), with an average of 22.47 µmol/L (Trolox). The highest DPPH radical scavenging capacity was observed in ‘Jiang Mi Guo Sang’ (29.82 µmol/L Trolox), which may be attributed to its elevated superoxide dismutase (SOD) (22.00 U·min·g^−^¹ FW) and peroxidase (POD) (10.14 U·min·g^−^¹ FW) activities. Previous studies have shown that catalase (CAT) and SOD enhance drought tolerance by detoxifying hydrogen peroxide (H_2_O_2_) and facilitating the conversion of superoxide radicals (SOR) to H_2_O_2_, respectively, suggesting that drought tolerance in plants can be regulated by controlling CAT and SOD activity (Umakanta and Shinya, 2018). The DPPH radical scavenging capacities of ‘Lv Shen Zi 1’, ‘Ju Shen’, ‘Lv Shen Zi 2’, ‘Xiao Bai E’, ‘Da Bai E’, ‘Tian Sang 202’, and ‘Hong Guo 1’ showed no significant differences but were significantly higher than those of other varieties. The lowest DPPH radical scavenging capacity was recorded in ‘Bai Shen 2’ (12.12 µmol/L Trolox).

The ABTS radical scavenging capacity ranged from 14.73 to 20.10 µmol/L (Trolox equivalents), with an average of 17.33 µmol/L (Trolox). The highest ABTS radical scavenging capacity was observed in ‘Lv Shen Zi 2’ (20.10 µmol/L Trolox), which was not significantly different from ‘Ju Shen’, ‘Lv Shen Zi 1’, ‘Su Bai Shen’, ‘Hei Zhen Zhu’, ‘Ri Ben Guo Sang’, and ‘He Lan Sang’ but was significantly higher than that of other varieties. The ABTS radical scavenging capacity of ‘Jiang Mi Guo Sang’ was not significantly different from ‘Feng Guo Sang’, ‘Da Ma Ya’, ‘Hong Guo 1’, ‘Tian Sang 202’, ‘Tang 10’, ‘Da Bai E’, ‘Da Yi Bai’, ‘Gui Hua Mi’, and ‘Lv Shen Zi’. The lowest ABTS radical scavenging capacity was observed in ‘Bai Shen 2’ (14.73 µmol/L Trolox).

The iron-reducing capacity, which reflects the ability to donate electrons and reduce Fe³^+^ to Fe²^+^, ranged from 71.07 to 255.08 µmol/L (Trolox equivalents), with an average of 126.20 µmol/L (Trolox). ‘Da Bai E’ exhibited the highest iron-reducing capacity (255.08 µmol/L Trolox), which was significantly higher than that of all other varieties.

The MDA content, an important biomarker of lipid peroxidation and oxidative stress, ranged from 20.46 to 74.89 µmol/g, with an average of 37.24 µmol/g. ‘Da 10’ exhibited the highest MDA content (74.89 µmol/g), which was significantly higher than that of all other varieties. ‘He Lan Sang’ had the second-highest MDA content (56.93 µmol/g), which was not significantly different from that of ‘Da Ma Ya’ and ‘Jiang Mi Guo Sang’ but was significantly higher than that of other varieties. The lowest MDA content was recorded in ‘Lv Shen Zi 2’ (20.46 µmol/g), which was not significantly different from ‘Feng Guo Sang’, ‘Lv Shen Zi 1’, ‘Lv Shen Zi’, ‘Xiao Bai E’, ‘Ri Ben Guo Sang’, and ‘Su Bai Shen’.

#### 3.4.2 Differences in enzyme activity of frost mulberry leaves among different varieties

The results of the measurements of superoxide dismutase (SOD), peroxidase (POD), catalase (CAT), polyphenol oxidase (PPO), and lipoxygenase (LOX) activities in the frost leaves of different mulberry varieties are presented in **Table 6**.

**Table 6:** Differences in the enzyme activity of mulberry leaf in different varieties of mulberry trees. (U·min·g^-1^FW).

Varieties	SOD activity	POD activity	CAT activity	PPO activity	LOX activity
Ri Ben Guo Sang	19.97 ± 0.78defg	16.92 ± 1.96bcd	36.33 ± 3.64cd	129.44 ± 11.66ab	168.00 ± 10.39de
Jiang Mi Guo Sang	27.17 ± 1.16a	14.35 ± 1.12de	39.50 ± 4.59bc	46.80 ± 5.77jk	124.00 ± 34.12e
Tian Sang 202	21.57 ± 0.81cde	19.08 ± 0.67ab	30.33 ± 6.48def	77.20 ± 19.8fghi	138.00 ± 43.27e
Hong Guo 1	23.23 ± 2.16bc	20.59 ± 1.66a	37.92 ± 2.92cd	81.76 ± 9.17efg	152.00 ± 81.46e
Bai Shen 2	18.74 ± 2.06efg	5.88 ± 1.80k	27.50 ± 1.52efg	75.28 ± 9.62fghij	258.00 ± 111.12bcd
Lv Shen Zi	20.58 ± 2.70cdefg	12.5 ± 0.52ef	34.67 ± 4.06cde	83.28 ± 8.66ef	176.00 ± 22.72de
Lv Shen Zi 1	19.57 ± 1.69efg	12.65 ± 2.52ef	69.58 ± 6.85a	119.20 ± 4.86bc	126.00 ± 15.87e
Lv Shen Zi 2	18.28 ± 1.64fg	9.14 ± 0.75hij	36.17 ± 3.26cd	140.24 ± 13.85a	284.00 ± 52.42bc
Hei Zhen Zhu	26.81 ± 1.16a	8.45 ± 1.22ij	12.25 ± 1.75ij	37.36 ± 8.93k	330.00 ± 49.11b
Ji Gui Hua	20.54 ± 2.04cdefg	8.88 ± 1.47hij	36.58 ± 2.67cd	55.84 ± 10.71ijk	204.00 ± 84cde
Gui Hua Mi	19.55 ± 2.35efg	12.53 ± 1.83ef	30.25 ± 3.44def	62.88 ± 7.28ghij	200.00 ± 54.11cde
Xiao Bai E	17.72 ± 0.28g	12.20 ± 1.00efg	30.75 ± 2.18def	60.00 ± 9.5hij	128.00 ± 12.49e
Da Bai E	21.6 ± 2.33cde	11.49 ± 1.13fgh	34.00 ± 7.60cde	65.68 ± 6.39fghij	174.00 ± 53.33de
Da Yi Bai	20.94 ± 1.36cdef	9.76 ± 0.94ghi	12.25 ± 3.61ij	99.20 ± 16.46de	140.00 ± 6.93e
Su Bai Shen	23.07 ± 1.35bcd	15.50 ± 2.63d	22.42 ± 3.79gh	53.84 ± 15.72jk	170.00 ± 56.71de
Feng Guo Sang	20.82 ± 1.86cdefg	10.21 ± 2.03fghi	19.33 ± 2.02hi	7.04 ± 3.00l	516.00 ± 102.53a
He Lan Sang	26.40 ± 1.26a	10.89 ± 0.53fghi	35.42 ± 4.54cde	122.56 ± 16.10ab	142.00 ± 24.25e
Ju Shen	25.08 ± 1.18ab	6.81 ± 0.76jk	10.75 ± 2.46j	60.80 ± 3.06hij	184.00 ± 30.79de
Da 10	24.93 ± 1.74ab	18.67 ± 1.02abc	45.83 ± 6.93b	57.44 ± 12.53hij	170.00 ± 55.75de
Tang 10	21.73 ± 1.41cde	16.26 ± 1.24cd	25.75 ± 3.25fgh	11.92 ± 3.50l	152.00 ± 15.1e
Da Ma Ya	18.31 ± 1.19fg	14.36 ± 1.36de	31.08 ± 3.50def	103.68 ± 8.65cd	122.00 ± 21.07e

Different lowercase letters indicate significant differences in Duncan test (P<0.05).

The SOD activity in frost mulberry leaves ranged from 15.39 to 22.68 U·min·g^−^¹ FW, with an average of 19.37 U·min·g^−^¹ FW. The highest SOD activity was observed in ‘Hei Zhen Zhu’ (22.68 U·min·g^−^¹ FW), which was not significantly different from ‘Lv Shen Zi’ but was significantly higher than that of other varieties. The SOD activity of ‘Ji Gui Hua’ was not significantly different from that of ‘He Lan Sang’, ‘Lv Shen Zi 2’, ‘Xiao Bai E’, ‘Da 10’, ‘Da Bai E’, ‘Su Bai Shen’, ‘Gui Hua Mi’, ‘Da Yi Bai’, and ‘Ju Shen’, but was significantly lower than that of other varieties (15.39 U·min·g^−^¹ FW).

Peroxidase (POD) activity is associated with enhanced resistance to chronic diseases, such as diabetes, cancer, and cardiovascular diseases, contributing to overall health benefits (Wang et al., 2021). The POD activity ranged from 1.89 to 14.11 U·min·g^−^¹ FW, with an average of 7.10 U·min·g^−^¹ FW. ‘Da 10’ exhibited the highest POD activity (14.11 U·min·g^−^¹ FW), which was not significantly different from that of ‘Hong Guo 1’ and ‘Da Yi Bai’, but was significantly higher than that of other varieties. The POD activity of ‘Hei Zhen Zhu’ was not significantly different from that of ‘Feng Guo Sang’, ‘Ju Shen’, ‘Ri Ben Guo Sang’, ‘Su Bai Shen’, ‘Lv Shen Zi’, ‘Xiao Bai E’, ‘Lv Shen Zi 1’, ‘Gui Hua Mi’, ‘Ji Gui Hua’, ‘Tang 10’, and ‘He Lan Sang’, but was significantly lower than that of other varieties (1.89 U·min·g^−^¹ FW).

Catalase (CAT) plays a crucial role in hydrogen peroxide detoxification, thereby mitigating oxidative damage and preventing cellular aging and tissue degeneration (Umakanta and Shinya, 2018). CAT activity ranged from 2.28 to 26.06 U·min·g^−^¹ FW, with an average of 7.10 U·min·g^−^¹ FW. ‘Tian Sang 202’ exhibited the highest CAT activity (26.06 U·min·g^−^¹ FW), which was not significantly different from that of ‘Jiang Mi Guo Sang’, ‘Bai Shen 2’, and ‘Gui Hua Mi’, but was significantly higher than that of other varieties.

The PPO activity, which influences enzymatic browning and plant defense mechanisms, ranged from 25.13 to 108.33 U·min·g^−^¹ FW, with an average of 55.90 U·min·g^−^¹ FW. ‘Da 10’ exhibited the highest PPO activity (108.33 U·min·g^−^¹ FW), which was not significantly different from that of ‘Da Yi Bai’ and ‘Da Bai E’, but was significantly higher than that of other varieties.

LOX activity, which plays a role in the biosynthesis of signaling molecules such as jasmonic acid, is crucial for plant responses to environmental stressors (Zhu et al., 2024). The LOX activity in frost mulberry leaves ranged from 79.58 to 532.08 U·min·g^−^¹ FW, with an average of 264.58 U·min·g^−^¹ FW. The highest LOX activity was observed in ‘Da Bai E’ (532.08 U·min·g^−^¹ FW), which was significantly higher than that of other varieties. The LOX activity of ‘Gui Hua Mi’ was not significantly different from that of ‘Da Yi Bai’. Similarly, ‘Bai Shen 2’, ‘He Lan Sang’, and ‘Da Ma Ya’ showed no significant differences in LOX activity. ‘Jiang Mi Guo Sang’ exhibited the lowest LOX activity (79.58 U·min·g^−^¹ FW), which was not significantly different from that of ‘Su Bai Shen’, ‘Ri Ben Guo Sang’, ‘Tang 10’, ‘Feng Guo Sang’, ‘Tian Sang 202’, ‘Gui Hua Mi’, ‘Lv Shen Zi 1’, ‘Lv Shen Zi 2’, and ‘Xiao Bai E’.

### 3.5 Comprehensive evaluation of the quality of frost mulberry leaves among different mulberry varieties

#### 3.5.1 Correlation analysis

**Figure 1:**
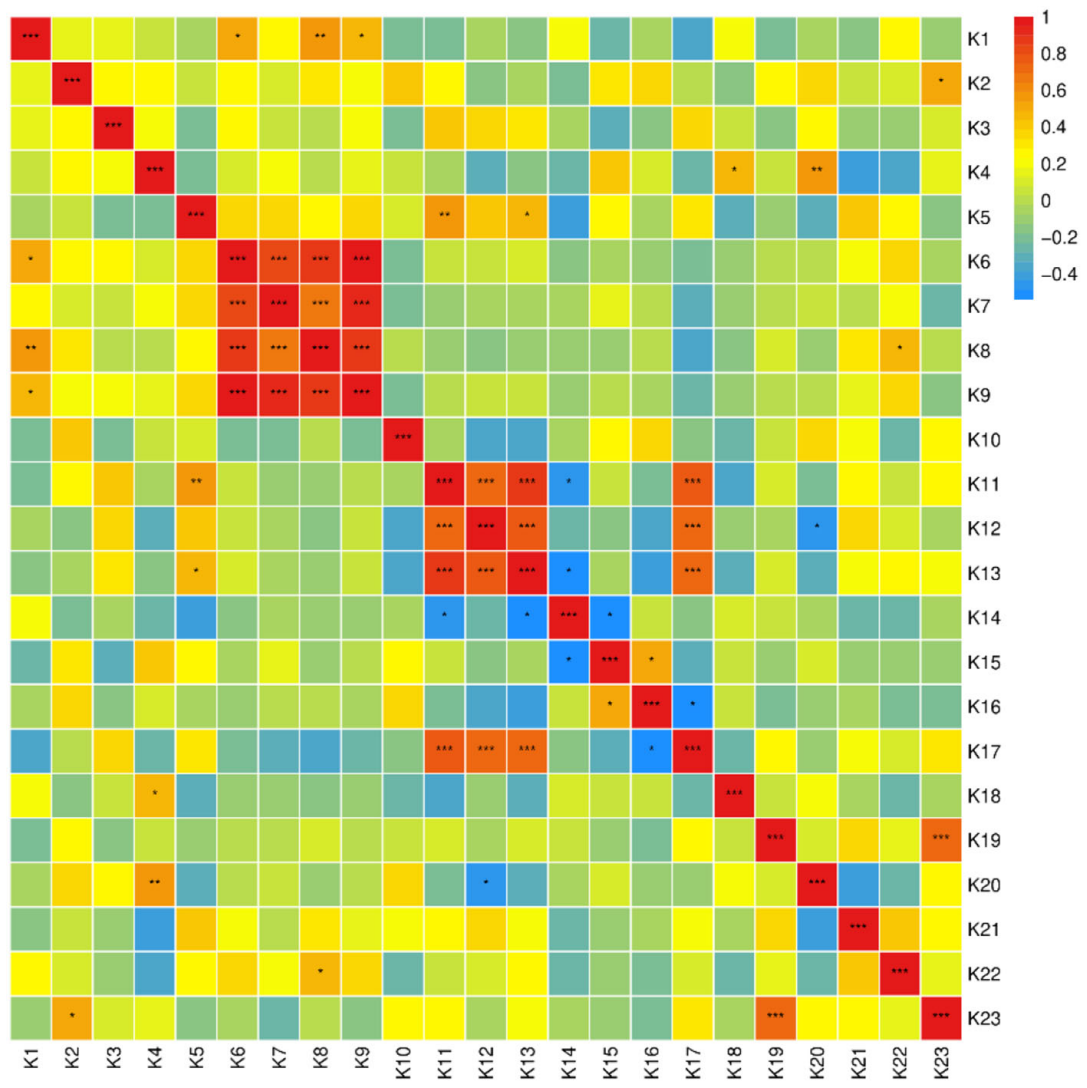
Correlation analysis of mulberry leaf quality traits. K1-water content, K2-free amino acid content, K3-soluble sugar content, K4-soluble protein content, K5-tannin content, K6-chlorophyll a content, K7-chlorophyll b content, K8-carotenoid content, K9-total chlorophyll, K10-flavonoid content, K11-polyphenol content, K12-resveratrol content, K13-chlorogenic acid content, K14-ascorbic acid content, K15-scavenging DPPH free radical capacity, K16-scavenging ABTS free radical Base capacity, K17—iron reducing ability, K18—SOD activity, K19—POD activity, K20—CAT activity, K21—PPO activity, K22—LOX activity, K23—MDA content. Same below. * indicates P<0.05,** indicates P<0.01, and *** indicates P<0.001.

Based on the Pearson correlation analysis (Pan et al., 2024) of 23 quality traits in frost mulberry leaves from 21 mulberry varieties (**Figure 1**), 16 pairs of indicators exhibited highly significant correlations (*P* < 0.01), while 12 pairs showed significant correlations (*P* < 0.05). However, the majority of indicators were not significantly correlated. Among the five nutritional quality indicators, no significant correlations were observed. Photosynthesis is a key physiological indicator of a plant’s synthetic function, and chlorophyll content is closely related to photosynthetic capacity (Riddhipratim and Gorachand, 2020). A higher chlorophyll content enhances sunlight absorption for photosynthesis, thereby improving mulberry leaf quality. Among the four photosynthetic pigment indicators, total chlorophyll content (K9) was significantly positively correlated with chlorophyll a content (K6), chlorophyll b content (K7), and carotenoid content (K8). Carotenoids play a dual role in photosynthesis: they complement chlorophyll in capturing light energy and protect it from photo-oxidation (Moise et al., 2014). Thus, an increase in total chlorophyll content is associated with higher carotenoid content. Among the five functional component indicators, polyphenol content (K11), resveratrol content (K12), and chlorogenic acid content (K13) exhibited highly significant positive correlations. However, ascorbic acid content (K14) showed a significant negative correlation with both polyphenol (K11) and chlorogenic acid (K13) content. Among the nine antioxidant activity indicators, POD activity (K19) was highly significantly positively correlated with MDA content (K23). Additionally, iron-reducing capacity (K17) was highly significantly positively correlated with polyphenol (K11), resveratrol (K12), and chlorogenic acid (K13) content. This finding suggests that phenolic compounds contribute to antioxidant activity, and higher levels of these compounds result in greater iron-reducing capacity. In summary, while certain correlations exist among the quality traits of frost mulberry leaves, these traits largely remain independent of one another.

#### 3.5.2 Principal component analysis

Principal Component Analysis (PCA) is a statistical technique used to identify the main components that explain the maximum variance in a dataset, thereby reducing dimensionality and focusing on the most critical variables affecting quality (Sun et al., 2018). Lou et al. (2024) applied PCA to analyze five mulberry varieties, demonstrating its effectiveness in selecting key evaluation indicators. To reduce the number of quality trait indicators of frost mulberry leaves from 23 to a smaller set of representative ones—while minimizing error and information loss—PCA was employed. Additionally, the entropy weight method (EWM) was used to assign weights, and grey relational analysis (GRA) was applied for comprehensive evaluation. PCA was conducted on 23 indicators across 21 mulberry varieties, extracting seven common factors (eigenvalues >1), as presented in **Table 7**, including variance contribution rates, cumulative variance contribution rates, and factor loadings. The cumulative variance contribution rate of these seven factors was 83.752%, indicating that they collectively explain 83.752% of the total variance, effectively representing the independent variables. This reduction from 23 indicators to 7 uncorrelated components enhanced interpretability while preserving most of the original information (Jolliffe and Cadima, 2016).

**Table 7:** Principal component eigenvalues and variance contribution rate of mulberry leaf quality of different varieties.

Component	Eigenvalues	Variance contribution rate/%	Accumulated variance contribution rate/%
1	4.820	20.955	20.955
2	4.422	19.228	40.183
3	2.883	12.536	52.719
4	2.430	10.567	63.286
5	2.219	9.646	72.931
6	1.383	6.011	78.942
7	1.106	4.809	83.752

Factor loadings reflect the relative influence of each indicator on the principal components, with positive values indicating a positive impact and negative values indicating a negative impact (**Table 8**). In the first common factor, the indicators with higher loadings and greater impact were chlorogenic acid content (0.859), polyphenol content (0.801), resveratrol content (0.790), iron-reducing capacity (0.703), and tannin content (0.634). The variance contribution rate of the first common factor was 20.955%, indicating the greatest impact on frost mulberry leaf quality. In the second common factor, the indicators with higher loadings and greater impact were total chlorophyll content (0.912), chlorophyll a content (0.897), carotenoid content (0.893), and chlorophyll b content (0.839). The variance contribution rate of the second common factor was 19.228%, which mainly explained the photosynthetic pigment content of frost mulberry leaves. Since total chlorophyll content better reflects pigment content compared to chlorophyll a, chlorophyll b, and carotenoid content, total chlorophyll content was selected as the representative indicator of the second common factor. In the third common factor, the indicators with higher loadings and greater impact were free amino acid content (0.776) and MDA content (0.622), with a variance contribution rate of 12.536%. In the fourth common factor, the indicator with a higher loading and greater impact was soluble sugar content (0.671), with a variance contribution rate of 10.567%. In the fifth common factor, the indicator with a higher loading and greater impact was POD activity (0.613), with a variance contribution rate of 9.646%. The variance contribution rates of the sixth and seventh common factors were 6.011% and 4.809%, respectively.

**Table 8:** Principal component factor loading matrix of mulberry leaves of different varieties.

Index	F1	F2	F3	F4	F5	F6	F7
Chlorogenic acid content	0.859	-0.271	0.073	0.081	-0.260	-0.053	-0.022
Polyphenol content	0.801	-0.287	0.343	0.033	-0.269	0.186	0.070
Resveratrol content	0.790	-0.275	-0.209	0.043	-0.248	0.005	0.259
Iron reducing ability	0.703	-0.575	0.077	0.259	0.004	0.061	-0.102
Tannin content	0.634	0.190	0.137	-0.498	-0.156	-0.068	-0.111
PPO activity	0.503	0.035	0.115	-0.296	0.492	-0.208	0.330
LOX activity	0.495	0.322	-0.065	-0.094	0.414	-0.104	0.032
Total chlorophyll content	0.330	0.912	-0.005	0.102	-0.071	-0.020	-0.107
Chlorophyll a content	0.380	0.897	0.014	0.140	-0.029	0.025	-0.029
Carotenoid content	0.232	0.893	0.017	0.006	0.274	0.060	0.028
Chlorophyll b content	0.172	0.839	-0.040	-0.005	-0.165	-0.130	-0.282
Water content	-0.019	0.599	-0.274	0.290	0.020	0.207	0.374
Free amino acid content	0.021	0.281	0.766	0.141	0.109	0.351	0.238
MDA content	0.148	-0.195	0.622	0.385	0.526	-0.063	0.063
Flavonoid content	-0.304	-0.068	0.595	-0.330	0.250	0.378	-0.064
Ability to scavenge DPPH free radicals	-0.174	0.115	0.551	-0.535	-0.429	-0.246	0.057
CAT activity	-0.424	0.062	0.499	0.490	-0.129	0.014	-0.351
Ascorbic acid content	-0.428	-0.045	-0.496	0.254	0.357	0.287	0.007
Soluble protein content	-0.352	0.207	0.489	0.375	-0.480	-0.314	0.026
Soluble sugar content	0.268	0.018	0.060	0.671	-0.364	0.379	0.192
Ability to scavenge ABTS free radicals	-0.447	0.161	0.257	-0.505	-0.144	0.268	0.421
POD activity	0.143	-0.106	0.392	0.274	0.613	-0.413	-0.027
SOD activity	-0.402	0.030	-0.094	0.328	-0.182	-0.566	0.511

The core evaluation indicators for the quality of frost mulberry leaves across 21 mulberry varieties were identified as chlorogenic acid content, polyphenol content, resveratrol content, iron-reducing capacity, tannin content, total chlorophyll content, free amino acid content, MDA content, soluble sugar content, and POD activity. These 10 indicators accounted for 83.752% of the total variance, effectively summarizing the original 23 traits while maintaining analytical robustness.

#### 3.5.3 Determination of evaluation indicator weights using the entropy weight method

Several methods can be employed to determine indicator weights, including the entropy weight method (EWM), analytic hierarchy process (AHP), preference ranking organization method for enrichment evaluation (PROMETHEE), and cost-benefit analysis. Among these, EWM was selected in this study to analyze the indicator weights. The entropy weight method is an objective weighting approach that quantifies the variation among observations for a given indicator. By fully capturing the intrinsic patterns and information content within the data, EWM minimizes subjectivity and randomness in weight calculation, ensuring a more data-driven and reliable evaluation (Tian and Wu, 2024).

When applying the entropy weight method (EWM) to determine factor weights, the quality evaluation data of frost mulberry leaves from 21 mulberry varieties were first standardized. Standardization is a crucial preprocessing step that eliminates unit and magnitude differences among indicators, ensuring data comparability. This standardized dataset provided a robust foundation for accurate weight calculations. Following the EWM computational process, factor weights were derived. Entropy values reflect the degree of variability in observed data: lower entropy values indicate greater differences among observations, signifying more informative indicators that play a larger role in comprehensive evaluation, resulting in higher assigned weights. The final weight calculation results are presented in **Table 9**. Among the 10 core evaluation indicators, iron-reducing capacity, POD activity, and resveratrol content exhibited the smallest entropy values, indicating higher difference coefficients and pronounced data gradients. Consequently, iron-reducing capacity was assigned a weight of 0.132, POD activity 0.122, and resveratrol content 0.119.

**Table 9:** Entropy weight method to determine the weight of 12 evaluation indicators.

Index	Entropy value	Coefficient of variance	Weights
Free amino acid content	0.919	0.081	0.106
Soluble sugar content	0.961	0.039	0.051
Tannin content	0.913	0.087	0.114
Total chlorophyll content	0.959	0.041	0.054
Polyphenol content	0.941	0.059	0.078
Resveratrol content	0.910	0.090	0.119
Chlorogenic acid content	0.915	0.085	0.111
Iron reducing ability	0.900	0.100	0.132
POD activity	0.907	0.093	0.122
MDA content	0.914	0.086	0.113

#### 3.5.4 Grey relational analysis method

Grey relational analysis (GRA), a fundamental component of grey system theory, has been widely applied in complex system analysis and multi-criteria evaluation across various disciplines in recent years (Jiang et al., 2025). GRA quantifies the degree of association between factors by comparing the similarity or dissimilarity of their development trends, expressed as the “grey relational degree.” This method enables the simultaneous evaluation of multiple quality attributes and ranks them based on their proximity to the optimal quality standards (Cao et al., 2024). According to grey system theory, the maximum values of all indicators among the tested mulberry varieties were designated as the optimal reference values to establish the reference sequence. The 10 core quality indicators of frost mulberry leaves from 21 mulberry varieties were normalized (dimensionless), and the absolute differences between observed and reference values were calculated. Subsequently, relational degree coefficients for each indicator were derived using the relational degree coefficient formula. Given that the importance of individual quality traits varies, the final grey relational degree was determined by incorporating the weights derived from the entropy weight method (EWM). The comprehensive evaluation results for frost mulberry leaf quality across different mulberry varieties are presented in **Table 10**. Based on these results, ‘Da Bai E’ (0.785), ‘Da Yi Bai’ (0.771), and ‘Da 10’ (0.717) exhibited the highest weighted relational degrees, indicating superior overall quality compared to other varieties.

**Table 10:** Weighted correlation degree and ranking of mulberry leaf quality of different varieties.

Varieties	Weighted relevance	Ranking
Da Bai E	0.785	1
Da Yi Bai	0.771	2
Da 10	0.717	3
He Lan Sang	0.684	4
Tang 10	0.674	5
Jiang Mi Guo Sang	0.659	6
Lv Shen Zi 1	0.623	7
Xiao Bai E	0.617	8
Ji Gui Hua	0.617	9
Bai Shen 2	0.602	10
Lv Shen Zi	0.599	11
Gui Hua Mi	0.578	12
Hong Guo 1	0.564	13
Ju Shen	0.550	14
Da Ma Ya	0.527	15
Ri Ben Guo Sang	0.516	16
Feng Guo Sang	0.504	17
Lv Shen Zi 2	0.502	18
Hei Zhen Zhu	0.500	19
Su Bai Shen	0.498	20
Tian Sang 202	0.476	21

## 4 Discussion

### 4.1 Quality of frost mulberry leaves among different mulberry varieties

Frost-treated mulberry leaves have long been recognized for their multifunctional properties. The quality of these leaves varies considerably among different cultivars and is influenced by genetic factors, environmental conditions, and cultivation practices. The evaluation of mulberry leaf quality typically involves a comprehensive assessment of multiple parameters, including photosynthetic pigment levels, nutritional quality, bioactive components, and antioxidant capacity.

#### 4.1.1 The photosynthetic pigment content of different varieties of frost mulberry leaves

Photosynthetic pigment levels influence the photosynthetic efficiency and are closely associated with the physiological activity of mulberry leaves. These pigments play a crucial role in photosynthesis, and their levels directly impact the photosynthetic efficiency, growth, and development of mulberry trees. Analyzing the photosynthetic pigment profiles of frost-treated leaves from different mulberry cultivars allows for a deeper understanding of their photosynthetic characteristics, offering a theoretical basis for cultivar selection and utilization. The synthesis and accumulation of photosynthetic pigments are also significantly affected by environmental factors and seasonal dynamics. Light intensity, light quality, temperature, water status, and soil nutrient availability could regulate the biosynthesis pathways of chlorophyll and carotenoids. Ivanov et al. (2020) studied the changes of chlorophyll a, chlorophyll b and carotenoid content in the leaves of 31 grassland and forest plants during the growing season, and found that the chlorophyll and carotenoid content per unit leaf mass of plants was related to environmental communities, geographical regions, and climatic conditions.

The dietary intake of carotenoids present in photosynthetic pigments is also vital for human health. For example, beta-carotene, a precursor of vitamin A, supports eye health and immune function. Lutein, a macular pigment, helps reduce the risk of age-related eye diseases (Sun et al., 2022). Ioana et al. (2024) conducted a study on the photosynthetic pigments of 12 basil (*Ocimum* spp.) cultivars and found that pigment differences were associated not only with nutritional value (carotenoids) and shelf life but also with processing traits (chlorophyll), serving as a potential quality indicator. Similarly, Zhang et al. (2012) analyzed the photosynthetic pigment and chlorophyll fluorescence characteristics of different strains of Porphyra variegata, concluding that regional origin may account for their differential temperature adaptability.

Analysis of 21 frost-treated mulberry leaf varieties revealed significant variation in photosynthetic pigment content. These variations directly influenced photosynthetic efficiency, antioxidant capacity, and biomass accumulation, ultimately affecting the quality and economic value of frost-treated mulberry leaves.

#### 4.1.2 The nutritional quality of different varieties of frost mulberry leaves

Young mulberry buds and leaves are nutrient-rich, containing protein, fiber, vitamins, and minerals (Sadowska and Bartosz, 2022; Chen C. et al., 2021). Nutritional quality, encompassing compounds such as soluble protein and soluble sugars, is a critical indicator for assessing the nutritional value of frost-treated mulberry leaves. The content of key nutrients such as soluble sugar, soluble protein, and DNJ in mulberry leaves is not static, and its accumulation process is highly sensitive to environmental conditions and seasonal changes. A study by Soltanabad et al. (2018) showed that the nutrient content in rosemary leaves is greatly influenced by the environment and precipitation. In this experiment, the soluble sugar content in frost-treated leaves across different mulberry cultivars ranged from 11.09% to 28.48%, with an average of 20.50%. The cultivar ‘He Lan Sang’ exhibited the highest soluble sugar content (28.48%). Meanwhile, soluble protein content ranged from 16.57 to 24.88 µg/g, with a mean of 19.82 µg/g. Among these, ‘Jiang Mi Guo Sang’ showed the highest soluble protein concentration.

Furthermore, mulberry leaves are additionally rich in bioactive compounds such as flavonoids and polyphenols. Most notably, their unique component, 1-deoxynojirimycin (DNJ), has been extensively demonstrated to possess hypoglycemic properties (Hu et al., 2013; Thakur et al., 2019). Numerous studies have confirmed that mulberry leaves function as an anti-diabetic food, as consuming mulberry leaves or their products can significantly reduce blood glucose levels.

Both nutrients and bioactive compounds exert synergistic effects that promote human health. Mulberry leaves have been identified as a valuable food resource, containing high levels of protein, carbohydrates, vitamins, trace elements, and dietary fiber, underscoring their rich nutritional profile (Butt et al., 2008; Srivastava et al., 2006). Therefore, in the production and promotion of mulberry leaves, nutritional value—alongside yield—is a key criterion in assessing mulberry leaf quality.

#### 4.1.3 The functional components of different varieties of frost mulberry leaves

The functional components, including polyphenols, flavonoids (He et al., 2020), polysaccharides (Chen X. L. et al., 2021), and alkaloids (Zhang et al., 2019), exhibit various pharmacological properties. These bioactive compounds have demonstrated medicinal value, contributing to antioxidant, anti-inflammatory, and other health-promoting effects (Fauzi et al., 2024). Consequently, frost mulberry leaves hold significant potential for applications in medicine and healthcare. The synthesis of polyphenols and flavonoids in plants is a secondary metabolic process, which is highly susceptible to environmental stress (such as UV radiation, low temperature, drought, pest infestation) and seasonal phenology. Cabrera et al. (2024) found that temperature has a greater effect on phenolic compounds when studying them. It can be inferred that the nutritional value of mulberry leaves after frost increases.

In recent years, with rising living standards and increasing health awareness, the dual-purpose value of mulberry leaves as both food and medicine has garnered significant attention. In the production and promotion of mulberry leaves, nutritional value is considered equally important as yield. The present study found that flavonoid content in frost mulberry leaves typically ranges from 53.15 to 120.04 mg/g. A higher flavonoid content is indicative of enhanced nutritional quality, making the leaves more suitable for medicinal applications. Flavonoids are a class of natural organic compounds widely distributed in plants, known for their diverse physiological activities, including antioxidant, anti-inflammatory, antiviral, and blood lipid-regulating effects. In frost mulberry leaves, an elevated flavonoid content suggests a greater potential for antioxidant activity and blood sugar regulation. For instance, extracts from mulberry leaves with high flavonoid concentrations may demonstrate greater efficacy in preventing cardiovascular diseases (Aghdam et al., 2024) and improving insulin sensitivity.

#### 4.1.4 The antioxidant qualities of different varieties of frost mulberry leaves

Padda and Picha (Padda and Picha, 2007) found that the antioxidant activity and chlorogenic acid content of mulberry leaves significantly increased after frost. Similar conclusions have been drawn by various researchers, indicating that frost-exposed mulberry leaves exhibit enhanced quality. Extensive experimental data confirm that frost mulberry leaves are rich in diverse antioxidant compounds, which neutralize free radicals, thereby delaying aging and enhancing immune function. Beyond flavonoids, frost mulberry leaves contain a variety of phenolic compounds, further contributing to their antioxidant activity. Bisma et al. (2022) reported significant differences in total phenol content and antioxidant activity among various mulberry leaf varieties. Similarly, Lee (2012) studied 13 mulberry leaf varieties from three regions and observed significant variations in both antioxidant and antibacterial activities across different varieties. In the present study, ‘Jiang Mi Guo Sang’ and ‘Da Bai E’ demonstrated notable antioxidant capacity, highlighting the potential of frost mulberry leaves as an appealing option for health-conscious consumers.

The enzyme activity in frost mulberry leaves plays a crucial role in metabolism, maturation, and medicinal properties (Son et al., 2019). Different enzymes contribute to distinct physiological functions in mulberry leaves. Among them, superoxide dismutase (SOD), catalase (CAT), and peroxidase (POD) serve as the primary antioxidant enzymes in mulberry leaves. SOD catalyzes the dismutation of superoxide anions into hydrogen peroxide and oxygen, thereby reducing free radical-induced cellular damage. CAT further decomposes hydrogen peroxide produced by SOD into water and oxygen, working in synergy with SOD to maintain reactive oxygen species (ROS) balance in mulberry leaf cells. POD facilitates the oxidation of various substrates using hydrogen peroxide, effectively removing peroxides and harmful phenolic compounds. This process helps mitigate oxidative stress and enhance mulberry leaves’ resistance to external stressors. Collectively, antioxidant enzymes contribute to preserving mulberry leaf quality, safeguarding their medicinal components, and thereby enhancing their therapeutic value. Additionally, polyphenol oxidase (PPO) catalyzes the oxidation of phenolic compounds in frost mulberry leaves. Since these leaves contain abundant phenolic compounds, PPO activity leads to their transformation into quinones, which can polymerize or react with other compounds, resulting in tissue darkening. This browning reaction is particularly evident during mulberry leaf maturation and is exacerbated when leaves sustain damage.

The quality of frost mulberry leaves is influenced by several factors, including the original growing environment, picking time, and processing and storage methods. Different mulberry varieties possess distinct advantages, which vary depending on their intended use and economic value. In recent years, the market demand for the medicinal properties of frost mulberry leaves has been increasing. To meet this growing demand, further research on optimal harvesting periods and effective processing and storage techniques is essential. Such studies can help preserve bioactive compounds, enhance medicinal efficacy, and improve overall product quality.

### 4.2 Comprehensive evaluation of frost mulberry leaves among different mulberry varieties

The nutritional, functional, and antioxidant properties of mulberry leaves are multifaceted, with multiple interrelated evaluation indicators. Conducting a correlation analysis of these indicators provides a scientific basis for selecting appropriate nutritional quality evaluation criteria. Given that different indicators have varying degrees of influence, applying a uniform weighting method would be scientifically inappropriate. Therefore, factor analysis serves as an effective tool to simplify and optimize evaluation parameters. Mulberry leaves are rich in diverse nutritional components and bioactive compounds, including proteins, mineral elements, vitamins, polysaccharides, polyphenols, flavonoids (He et al., 2020), and alkaloids (Zhang et al., 2018). These compounds exhibit various pharmacological effects, such as lipid-lowering, blood sugar regulation (Liu et al., 2012; Han et al., 2020), fatty liver prevention, anti-tumor activity (Park et al., 2013), anti-aging properties (Hacer et al., 2016), and anti-inflammatory effects. Alkaloids and polysaccharides are the primary active components responsible for the hypoglycemic effect (Asano et al., 1994). First, the alkaloids in mulberry leaves have a unique structure that allows them to replace sugar and bind to α-glucosidase, showing strong inhibitory activity with low toxicity. This action inhibits the breakdown of polysaccharides in the body and reduces the production of new glucose (Asano et al., 2002; Taniguchi et al., 1998). Second, compounds found in mulberry leaves, such as those from a buckwheat base, stimulate the islet beta cells, promoting the secretion of insulin. This, in turn, facilitates the breakdown of glucose in the blood and the synthesis of liver glycogen while inhibiting the rise of blood sugar (Chen et al., 2018; Cui et al., 2023).

The National Health Commission has officially recognized mulberry leaves as a “dual-purpose” plant (He et al., 2018), suitable for both food and medicinal applications. Furthermore, mulberry leaf extracts contain a range of bioactive compounds, including flavonoids, γ-aminobutyric acid, 1-deoxynojirimycin, and chlorophyll (Vichasilp et al., 2009), highlighting their broad therapeutic potential.

In the comprehensive evaluation of 21 mulberry varieties, key indicators—such as chlorogenic acid content—account for 83.75% of the core evaluation criteria, effectively reflecting both medicinal and nutritional components. These indicators play a crucial role in identifying high-quality mulberry varieties, facilitating their development and application. This study provides a scientific basis for mulberry variety selection in Hebei Province by conducting a comprehensive analysis of mulberry leaf quality across multiple varieties. To identify the main evaluation indicators, correlation analysis (CA) and principal component analysis (PCA) were employed. Additionally, the entropy weight method (EWM) and grey relational analysis (GRA) were used to determine the most suitable high-quality varieties for the local environment based on comprehensive evaluation scores. The comprehensive analysis provides objective evaluation results, serving as a scientific basis for screening high-quality edible mulberry varieties, refining the evaluation system, guiding the selection of specialized varieties, optimizing resource utilization, and enhancing mulberry leaf product development.

Principal component analysis (PCA) standardizes and analyzes fruit and leaf quality traits through dimension-reduction techniques. It calculates principal factor values as weights based on their variance contribution rates and determines scores by dividing the cumulative sum of the products of these values and the initial eigenvalues of corresponding principal components by the sum of initial eigenvalues (Vinti et al., 2020). Abdollah et al. (2014) employed PCA to study 16 fruit parameters across 23 grape varieties, revealing extensive fruit diversity within the grape germplasm, with key discriminative parameters associated with berry size. Similarly, Fan et al. (2020) analyzed the primary nutritional components of 23 wild Corylus heterophylla varieties in Northeast China to identify superior selections and examine factors influencing nut quality. Zhang et al. (2024) utilized PCA to compare 14 indicators across 11 pitaya varieties in Guangzhou, China, for comprehensive evaluation and ranking, concluding that red-skinned and red-fleshed pitayas exhibit longer fruiting periods and higher yield potential. PCA effectively eliminates correlations among recombined indicators, enhancing the interpretability of complex trait dataset. In order to identity high quality mulberry leaves that are suitable for healthy products to expand planting, 24 samples from three regions (Guangdong, Guangxi, Chongqing) in the south of China were quantified for two alkaloids (1-deoxynojirimycin and fagomine) and five phenols (chlorogenic acid, rutin, isoquercitrin, etc.) using high-performance liquid chromatography tandem mass spectrometry (HPLC-MS/MS). Additionally, the total phenolic and total flavonoid contents, antioxidant and glycosidase inhibitory activities (hypoglycemic activity) were tested using different assays (DPPH, ABTS, FRAP) to comprehensively evaluate the quality of the mulberry leaves (Hao et al., 2018).

This study integrates multiple analytical methods to comprehensively evaluate mulberry leaf quality across different varieties, providing a scientific foundation for mulberry variety selection in Hebei Province. The findings aim to guide variety selection, breeding programs, and quality control strategies in commercial production. However, this study primarily focuses on mulberry leaves, while the potential value of mulberry buds and other plant parts remains underexplored. Future research will expand the scope of evaluation to establish a more comprehensive and rigorous scientific assessment system.

## 5 Conclusion

To comprehensively evaluate the primary mulberry varieties in Hebei Province, frost mulberry leaves from 21 mulberry varieties were assessed based on nutritional quality, functional components, antioxidant capacity, and enzyme activity using principal component analysis (PCA) and grey relational analysis (GRA). The results revealed significant differences in quality attributes across varieties. Through PCA, 10 core evaluation indicators were identified, and the entropy weight method (EWM) was applied to determine their relative importance. Following grey system theory, data were standardized (dimensionless), and correlation degree coefficients were calculated using the correlation degree formula. The top five varieties by comprehensive evaluation scores were Da Bai E (0.785), Da Yi Bai (0.771), Da 10 (0.717), He Lan Sang (0.684), and Tang 10 (0.674).

Among the 21 tested mulberry varieties, ‘Da Bai E’ achieved the highest comprehensive score, demonstrating superior quality and high value. Its composition included 21.14% soluble sugar, 53.37% moisture, 128.51 mg/g free amino acids, 17.89 µg/g soluble protein, 2.38 mg/g tannins, 80.92 mg/g flavonoids, 111.44 mg/g polyphenols, 2.27 mg/g resveratrol, 50.87 mg/g chlorogenic acid, and 55.25 mg/100g ascorbic acid. This study provides a scientific basis for the selection, cultivation, and promotion of mulberry varieties in Hebei Province. The significant differences in quality attributes among the 21 varieties suggest that: Varieties with high nutritional quality are suitable for mulberry leaf tea and food products. Varieties rich in functional components can be utilized for medicinal applications. These findings expand the potential processing and utilization pathways for frost mulberry leaves, offering valuable insights for future product development and industrial applications.

## Data Availability

The raw data supporting the conclusions of this article will be made available by the authors, without undue reservation.
